# The Old Way and the New

**Published:** 1886-06

**Authors:** 


**The old way and the new.**—The old injunction to starve a fever and stuff a cold followed for many centuries as containing the quintescence of human wisdom, contained an error of great magnitude. Countless thousands of fever-stricken victims were offered as a sacrifice to this idea of starvation. A cold is a moderate fever ; if stuffing was good practice, then starvation in any fever was wrong. Experiment has shown the truth of this inference, and Graves, the great Dublin physician, was right when he desired no nobler epitaph than "He fed fevers." Systematic support by food given as medicine and by alcohol in some form—also as a food—forms now the most important element in the management of all the self limited diseases like typhus, typhoid, relapsing and yellow fever, pneumonia, consumption, dysentry and diptheria, the eruptive fevers and acute inflammations generally.

The reduction of excessive bodily heat being one of the most important ends to be reached by medical treatment, and quinine and its congeners not being always available, other means have been sought for and obtained for attaining the same object. The old styles of keeping the sick room hot and without fresh air, and covering the sick man with heavy non-conductors of heat, and not allowing him cold drinks, have been abandoned by every practitioner, whether he calls himself "regular," homœopathic, or eclectic. Cold baths, cooling drinks, ice and good ventilation are recognized as among the most efficient aids by the physician. The people are themselves learning some facts regarding the hygiene of the sick room that will render the old practices impossible in the near future.—*Medical Summary.*

